# Interventions to promote critical health literacy: a scoping review

**DOI:** 10.1093/heapro/daag103

**Published:** 2026-08-05

**Authors:** Martin Kalteis, Anke Steckelberg, Sandro Zacher, Jana Hinneburg

**Affiliations:** Institute of Health, Midwifery and Nursing Science, Medical Faculty of Martin Luther University Halle-Wittenberg, University Medicine Halle, Magdeburger Straße 8, Halle (Saale) 06112, Germany; Institute of Health, Midwifery and Nursing Science, Medical Faculty of Martin Luther University Halle-Wittenberg, University Medicine Halle, Magdeburger Straße 8, Halle (Saale) 06112, Germany; Institute of Health, Midwifery and Nursing Science, Medical Faculty of Martin Luther University Halle-Wittenberg, University Medicine Halle, Magdeburger Straße 8, Halle (Saale) 06112, Germany; Institute of Health, Midwifery and Nursing Science, Medical Faculty of Martin Luther University Halle-Wittenberg, University Medicine Halle, Magdeburger Straße 8, Halle (Saale) 06112, Germany

**Keywords:** critical health literacy, critical health competence, critical thinking, health literacy, health intervention, scoping review

## Abstract

Critical health literacy (CHL) is regarded essential for critically appraising health information and making informed decisions. However, evidence on CHL-promoting interventions is low, leaving unclear how such interventions are implemented and evaluated, and which conceptualizations of CHL they draw on. This scoping review systematically identified and characterized CHL interventions, including target populations and settings, theoretical and methodological underpinnings, CHL dimensions addressed, evaluation approaches and reported implementation barriers and facilitators. We searched MEDLINE, CINAHL, APA PsycInfo, PSYNDEXplus and ERIC to September 2025 and supplemented this with backward citation searching. Publications reporting interventions with a stated or implicit CHL focus were included and synthesized narratively. Database searches yielded 3715 records and citation searching identified 117 additional records. After screening, 81 publications describing 53 distinct interventions were included. Publication activity increased from 2016 onwards, peaking between 2021 and 2025. Interventions were conducted in 19 countries and were concentrated in educational settings, mostly targeting school and university students. Most interventions focused on appraisal skills and individual action (e.g. informed decision-making), while broader CHL dimensions such as understanding social determinants of health and collective action were rarely addressed. Barriers included limited time and resources, technical infrastructure constraints and high demands on teachers, while facilitators included leadership support, training or coaching and high-quality learning materials. To strengthen CHL’s preventive potential in health promotion, future interventions should prioritize large-scale effectiveness and follow-up studies, while accounting for context-sensitive adaptation, implementation conditions and broader operationalizations of all CHL dimensions across settings.

Contribution to Health Promotion

- Critical health literacy (CHL) helps people to evaluate health information, understand how social and living conditions shape health and act on health issues individually and with others.
- This scoping review maps 53 interventions across countries and settings that aim to strengthen CHL.
- Interventions predominantly addressed appraisal and individual decision-making, whereas social determinants and collective action were rarely integrated.
- Key implementation barriers were limited time and resources as well as an insufficient technical infrastructure. Facilitators included training programmes for deliverers, structured materials and organizational support.
- The findings inform the development and implementation of more comprehensive and equity-oriented CHL interventions.

## Introduction

Critical thinking is widely recognized as a core educational goal of the 21st century and a pre-requisite for active citizenship (OECD 2019). During crises such as the SARS-CoV-2 pandemic, rapidly circulating information can shape individual behaviour and public responses, while simultaneously increasing exposure to misinformation (Rovetta and Bhagavathula 2020, Tsao *et al*. 2021). Although quality criteria have been defined (such as Good practice guidelines for health information, 2016) and the *Guideline Evidence-based Health Information* (Lühnen *et al*. 2017) calls for transparency, systematic evidence retrieval and clear communication of benefits and harms, a lot of printed and online health information still falls short (Osman *et al*. 2022, Zacher *et al*. 2025, 2026, Kasper *et al*. 2026). Analyses of health websites repeatedly show limited comprehensibility and selective or biased presentation of key information (Zhang *et al*. 2015, Daraz *et al*. 2019). In this context, the ability to critically assess the trustworthiness of health information becomes essential (Tangcharoensathien *et al*. 2020, Borges do Nascimento *et al*. 2022).

Limited health literacy (HL) is common even in high-income countries such as the USA, UK, Germany and Switzerland. In population-based surveys conducted in these countries, nearly half of the participating adults report difficulties in understanding and applying health information in everyday life. This challenge is even more pronounced in relation to critical information appraisal, where around two-thirds of the respondents report difficulties (Public Health England and UCL Institute of Health Equity 2015, de Gani *et al*. 2021, Schaeffer *et al*. 2021, Ringle *et al*. 2025). Lower HL has been associated with higher hospitalization rates, more frequent emergency care use, lower uptake of preventive services and vaccinations, reduced medication adherence and poorer comprehension of medicine labels and health information. Among older adults, limited HL is also related to poorer health status and increased mortality (Berkman *et al*. 2011). HL is also shaped by cultural and situational demands and depends on equitable access to education and lifelong learning opportunities (Nutbeam and Muscat 2021).

Against this background, critical health literacy (CHL) is often discussed as a potential lever for improving health equity and participation. However, empirical evidence on the association between CHL and downstream outcomes, including health behaviours, remains heterogeneous and not unidirectional. While some studies report beneficial associations, others show mixed or context-dependent patterns, and a few also indicate negative associations. This suggests that the current evidence base remains too limited to support firm conclusions about the role of CHL in shaping health behaviours (Færevaag *et al*. 2026).

Accordingly, CHL may be less adequately captured by behavioural outcomes alone and more appropriately understood in relation to people’s capacities to critically engage with health information and make informed health-related decisions. CHL therefore involves higher-order cognitive and social capacities that enable people to critically analyze information and use it to gain greater control over health-related decisions and life circumstances (Nutbeam 2000). Benkert and Abel (2023) define CHL as the ability of an individual or a community to reflect on health-promoting factors and processes and to use the outcomes of this reflection for health-promoting individual and collective action. Building on this definition, they specify three core components: critically appraising health information, understanding the social determinants of health, and the capacity for individual and collective action (Benkert and Abel 2023). At an individual level, the action component of CHL involves the ability to critically appraise information in order to make informed decisions and meaningfully participate in shared decision-making (Muscat *et al*. 2021). CHL has also been linked to scientific literacy, understood as the capacity to use scientific knowledge critically, reflect on science-related issues and draw evidence-informed conclusions (OECD 2012). At the same time, no universally accepted definition of HL exists to date (Islertas 2022). Beyond HL frameworks, CHL has also been linked to scientific literacy, health information literacy, media literacy, evidence-based practice and critical thinking (Chinn 2011, Ringle *et al*. 2025). These concepts can therefore be understood as important, yet partial, components of CHL. Individually, however, they do not fully capture the conceptual breadth and functional scope of CHL.

Promoting CHL is thus of high relevance at both individual and societal levels. However, two important gaps remain: there is limited clarity regarding which competencies constitute CHL and limited evidence on how these can be effectively fostered. Accordingly, few interventions are explicitly designed to promote CHL (de Wit *et al*. 2017, Sykes and Wills 2018, Rubinelli *et al*. 2024). For instance, Stormacq *et al.* (2020) reported that HL interventions rarely build skills to assess the credibility and quality of health information, especially among disadvantaged groups. Similarly, Nutbeam *et al.* (2018) noted that intervention objectives are often not clearly defined and tend to target functional and interactive HL rather than CHL-specific content.

### Objectives

The aim of this scoping review was to systematically identify and characterize interventions intended to promote CHL. The review focused in particular on interventions with an explicit link to health information, given that the critical appraisal was considered a necessary aspect of developing CHL. We examined target populations and settings, the theoretical and methodological frameworks underpinning intervention development and implementation, the CHL dimensions addressed, evaluation approaches and reported facilitators and barriers to implementation.

The review questions (RQ) were

RQ1: Which interventions have been developed and implemented to promote CHL and in which target groups and settings?
  RQ1a: Which theoretical underpinnings and frameworks have informed the development?
  RQ1b: Which educational goals are employed in these interventions?
  RQ1c: Which educational and implementational strategies are used for CHL interventions?
  RQ1d: Which dimensions of CHL are addressed by these interventions?
  RQ1e: To what extent have the interventions been evaluated and what approaches or measures have been used to assess their outcomes?
RQ2: Which barriers and facilitators related to promoting CHL are reported?

## Methods

A scoping review was selected because the evidence base is conceptually diverse and spread across multiple disciplines, making an exploratory mapping of intervention types and contexts appropriate. The scoping review was conducted in accordance with current Joanna Briggs Institute (JBI) guidance for scoping reviews (Peters *et al*. 2020, Aromataris *et al*. 2024). Reporting follows the Preferred Reporting Items for Systematic reviews and Meta-Analyses extension for Scoping Reviews (PRISMA-ScR) (Tricco *et al*. 2018). Citation searching was reported in line with the Terminology, Application, and Reporting of Citation Searching (TARCiS) statement (Hirt *et al*. 2024). An *a priori* protocol was developed and registered on the Open Science Framework (OSF) in September 2025 (Kalteis *et al*. 2025). This study was based exclusively on literature. Therefore, no ethics approval was required.

### Eligibility criteria

To determine eligibility, we applied the population-concept-context framework as recommended for scoping reviews (Peters *et al*. 2020). We included studies involving any population group, without restrictions by age, gender, health status, socioeconomic position, education or cultural background. Eligible evidence sources reported interventions, programmes or strategies that were explicitly promoting CHL or addressed one or more CHL dimensions proposed by Benkert and Abel (2023), including critical appraisal of health information, understanding social determinants of health, or enabling individual and collective action. Given the conceptual overlap between CHL and related constructs, interventions were assessed according to their content rather than their label. Inclusion was based on the extent to which intervention objectives or content aligned with one or more CHL dimensions. We included all settings and delivery environments without restrictions by geography or institutional context (e.g. schools, higher education, healthcare organizations, community settings or digital environments). No restrictions were applied regarding publication year or language. For records not published in English or German that appeared potentially eligible, we used machine translation (DeepL Translate SE, 2025) to support screening and data charting. We included primary empirical research of any design and relevant grey literature, provided sufficient information was available to characterize the intervention and its evaluation. We excluded systematic reviews, meta-analyses and other review formats to avoid secondary selection and potential double counting of primary studies. We also excluded conference abstracts, blogs and media reports due to insufficient methodological detail for mapping intervention characteristics and evaluation.

### Search strategy

The search aimed to identify both published and unpublished evidence and followed the JBI-recommended nine step approach (Peters *et al*. 2020, Aromataris *et al*. 2024). First, we conducted an initial limited search in MEDLINE (via PubMed) to identify relevant records and to analyze keywords in titles, abstracts and associated indexing terms. Secondly, we developed a comprehensive search strategy built around two search components: (i) CHL, including related terminology and (ii) interventions, capturing programmes, educational strategies, training formats and implementation activities aimed at fostering CHL. The search strategy was adapted to each database (see Supplementary File S1).

The systematic database search was conducted up to 26 September 2025 in MEDLINE (PubMed), CINAHL (EBSCO), APA PsycInfo (Ovid), PSYNDEXplus (Ovid) and ERIC (IES). These databases were selected to reflect the multi-disciplinary nature of CHL interventions across medicine, public health, nursing and health services research, psychology and the social sciences and education research. To enhance search quality and reproducibility, the MEDLINE search strategy and its adaptations were reviewed using the PRESS guideline (McGowan *et al*. 2016).

Grey literature searching was conducted to mitigate publication bias and identify evidence not indexed in bibliographic databases. We searched doctoral theses and dissertations in the electronic theses online service and open access theses and dissertations and searched OpenGrey. As OpenGrey was discontinued in 2021, searches were conducted using its archived catalogue.

Given the field’s terminological heterogeneity and inconsistent indexing, we complemented database and grey literature searches with backward citation searches and manually checking reference lists. Backward citation searching was conducted between 29 October 2025 and 10 November 2025. Seed references (see Supplementary File S1) comprised all included full texts identified through database searching and additional relevant evidence syntheses identified during screening. All potentially relevant records identified through backward citation searching entered the same screening workflow as database-derived records.

### Study selection process

All records identified through database searching, grey literature searches and citation searching were imported into Citavi 6 (Swiss Academic Software, Zurich, Switzerland) and duplicates were removed. Screening was conducted using Rayyan (Ouzzani *et al*. 2016). Prior to formal screening, the review team piloted the eligibility criteria on a subset of records to calibrate interpretation and ensure consistent application.

Two reviewers (M.K. and J.H. or S.Z.) independently screened titles and abstracts. Full texts were obtained for all records considered potentially eligible and were assessed independently by two reviewers against the eligibility criteria. Records without an abstract were retained for full-text screening to minimize erroneous exclusions due to incomplete bibliographic information. Disagreements at any stage were resolved through discussion and, if required, consultation with a third reviewer (A.S.).

### Data extraction and analysis

We developed a structured data extraction template in Microsoft Excel and an accompanying extraction guide to support consistent charting. Data were extracted by one reviewer (M.K.) and independently checked by at least one other reviewer (J.H. or S.Z.) using a structured data extraction template in Microsoft Excel. Discrepancies were resolved through discussion and, if required, consultation with a third reviewer (A.S.). Disagreements concerning the assignment of CHL dimensions primarily occurred in cases where interventions did not explicitly refer to CHL concepts. All disagreements were resolved through discussion and consensus of all authors.

Extracted data were organized in three domains: First, we captured study and sample characteristics, including author and year, country, study design and population. Secondly, we charted intervention-specific information, including intervention name and focus, setting, delivery mode, educational and implementation strategies, theoretical or methodological underpinnings, implementation features, evaluation approaches and measures. Thirdly, we extracted reported barriers and facilitators related to intervention implementation.

To support consistent classification across heterogeneous interventions, we mapped targeted outcomes and content to CHL dimensions using the component model proposed by Benkert and Abel (2023), distinguishing critical appraisal of health information, understanding of social determinants of health, and individual and collective action.

Synthesis followed a descriptive approach appropriate for scoping reviews (Tricco *et al*. 2018, Peters *et al*. 2020). We summarized extracted data using frequency counts and structured narrative synthesis aligned with the review questions. We tabulated intervention characteristics, target populations and settings, delivery formats and evaluation approaches. Reported barriers and facilitators were collated across interventions and synthesized narratively.

## Results

Results are reported narratively and structured according to the review questions. Given the volume of included publications, findings are synthesized and condensed to maintain readability. Where multiple publications described the same intervention, these were consolidated at the intervention level and assigned a shared intervention identification number (I-ID). Multiple coding was permitted where interventions addressed more than one aspect of data charting.

**Figure 1:**
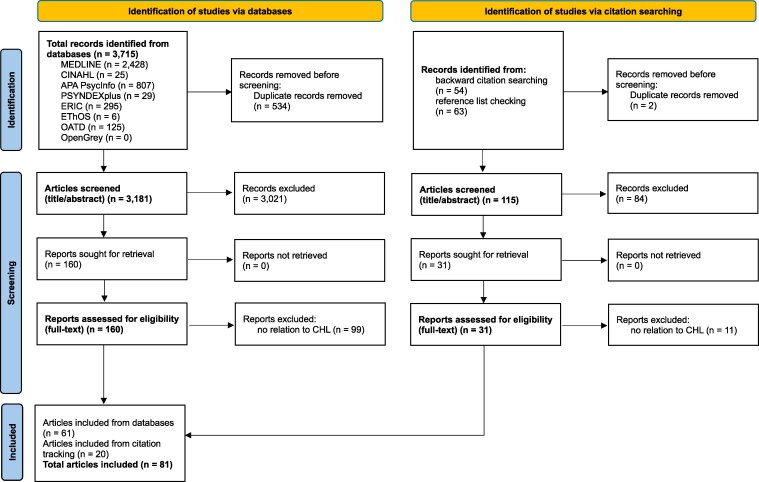
PRISMA flow diagram (adapted from Page *et al.* 2021).

Database searches yielded 3715 records and citation searching identified another 117. After screening, 81 studies were included, describing 53 distinct interventions. The list of excluded records is provided in Supplementary File S1. The study selection process is shown in the PRISMA flow diagram (Fig. 1 adapted from Page *et al.* 2021).

RQ1: Which interventions have been developed and implemented to promote CHL and in which target groups and settings?

### Characteristics of included articles

Across the 53 interventions, a total of 20 956 participants were reported. Interventions were conducted in 19 countries, most frequently in the USA (*n* = 17), followed by Germany (*n* = 8). Five interventions were conducted in Norway and four interventions each were conducted in Australia and Taiwan. Two interventions were conducted in each of Uganda, Kenya, Rwanda, Ireland, New Zealand, Canada and UK, while single interventions were located in Italy, Spain, Austria, Brazil, France, China and Malaysia. A complete overview of all included sources and their allocation to I-IDs is provided in Table 1.

**Table 1:** Characteristics of included studies.

I-ID:	Author, year:	Country	Study design	Intervention	Teaching/learning format	*N*	Target group	Setting
I-01	Nsangi *et al*. 2020b	Norway, Uganda, Kenya, Rwanda	Development study	IHC intervention for primary schools	Live	N/A	Students Teachers	Primary school
	Nsangi *et al*. 2017	Uganda	cRCT	IHC intervention for primary schools	Live	120 clusters 10 183 students 152 teachers	Students Teachers	Primary school
	Nsangi *et al*. 2020a	Uganda	Follow-up cRCT	IHC intervention for primary schools	Live	120 clusters 6787 students 143 teachers	Students Teachers	Primary school
	Nsangi *et al*. 2019	Uganda	Process evaluation	IHC intervention for primary schools	Live	60 clusters 84 teachers	Students Teachers	Primary school
I-02	Semakula *et al*. 2019a	Uganda	Development study	IHC podcast	Online, asynchronous	N/A	Parents Participants: researchers, journalists, parents, health communication professionals, media editors, radio producers, actors, musicians	Primary school
	Semakula *et al.* 2017	Uganda	RCT	IHC podcast	Online, asynchronous	675 parents	Parents	Primary school
	Semakula *et al*. 2020	Uganda	Follow-up RCT	IHC podcast	Online, asynchronous	523 parents	Parents	Primary school
	Semakula *et al*. 2019b	Uganda	Process evaluation	IHC podcast	Online, asynchronous	N/A	Parents	Primary school
I-03	Alderighi *et al*. 2022	Italy	Pilot study	IHC intervention for primary schools	Live	46 students 2 teachers	Students Teachers	Primary school
	Rasoini *et al*. 2025	Italy	Mixed-method-pilot study	IHC intervention for primary schools	Live	133 students 8 teachers	Students Teachers	Primary school
I-04	Jofra *et al*. 2023	Spain	Mixed-method-pilot study	IHC intervention for primary schools	Live	143 students 6 teachers	Students Teachers	Primary school
I-05	Rosenbaum *et al*. 2023	Norway, Uganda, Kenya, Rwanda	Development study	IHC intervention for secondary schools	Live	N/A	Students Teachers	Secondary school
	Chesire *et al*. 2023	Kenya	cRCT	IHC intervention for secondary schools	Live	80 clusters, 3362 students 80 teachers	Students Teachers	Secondary school
	Chesire *et al*. 2025	Kenya	Follow-up cRCT	IHC intervention for secondary schools	Live	80 clusters, 2446 students 67 teachers	Students Teachers	Secondary school
	Chesire *et al*. 2024	Kenya	Process evaluation	IHC intervention for secondary schools	Live	96 students 110 teachers 18 parents 14 others	Students Teachers Participants: parents/guardians, decision-makers, school leadership	Secondary school
	Mugisha *et al*. 2023	Rwanda	cRCT	IHC intervention for secondary schools	Live	84 clusters, 3199 students 89 teachers	Students Teachers	Secondary school
	Mugisha *et al*. 2025	Rwanda	Follow-up cRCT	IHC intervention for secondary schools	Live	84 clusters, 2419 students 70 teachers	Students Teachers	Secondary school
	Mugisha *et al*. 2024	Rwanda	Process evaluation	IHC intervention for secondary schools	Live	110 students 10 teachers 5 parents 12 others	Students Teachers Participants: parents/guardians, decision-makers, school leadership	Secondary school
	Ssenyonga *et al*. 2023	Uganda	cRCT	IHC intervention for secondary schools	Live	80 clusters 4853 students 80 teachers	Students Teachers	Secondary school
	Ssenyonga *et al*. 2025	Uganda	Follow-up cRCT	IHC intervention for secondary schools	Live	80 clusters, 3433 students 75 teachers	Students Teachers	Secondary school
	Ssenyonga *et al*. 2024	Uganda	Process evaluation	IHC intervention for secondary schools	Live	103 students 10 teachers 11 parents 13 others	Students Teachers Participants: parents/guardians, decision-makers, school leadership	Secondary school
	Oxman *et al*. 2025	Uganda, Kenya, Rwanda	Qualitative evaluation	IHC intervention for secondary schools	Live	30 students 2 teachers 8 parents 39 others	Students Teachers Participants: parents/guardians, stakeholders, school leadership	Secondary school
I-06	Deliv *et al*. 2023	Ireland	Development study	Animated video on evidence syntheses	Online, asynchronous	14 students	Students Teachers	Internet
I-07	Oxman *et al*. 2021	Norway	Case study	Evidence-based practice course in healthcare	Live	60	University students of health science	Higher education
	Elvsaas *et al*. 2024	Norway	Mixed-method-study	Evidence-based practice course in healthcare	Live	446	University students of health science	Higher education
I-08	Elvsaas *et al*. 2023	Norway	Mixed-method-study	Digital IHC game application (serious game)	Online, asynchronous	193	University students	Online
I-09	Li *et al*. 2025	Ireland	Development study	IHC online cancer learning resource	Online, asynchronous	33	Cancer patients, health professionals and researchers	Higher education
I-10	Austvoll-Dahlgren *et al*. 2012	Norway	RCT	Web portal for evidence-based information and HL	Online, asynchronous	96	Parents	Internet
	Austvoll-Dahlgren *et al*. 2013	Norway	Development study	Web portal for evidence-based information and HL	N/A	N/A	Public	Internet
I-11	Steckelberg *et al*. 2009a, 2009b	Germany	Pilot study	EbM course	Live	45	Students	Secondary school
I-12	Meyer *et al*. 2007	Germany	Pilot study	EbM course	Live	121	Diabetes educators	Diabetes centre
I-13	Berger *et al*. 2010	Germany	Pilot study	EbM course	Live	161	Self-help groups, patients and representatives	Higher education
	Berger *et al*. 2013	Austria	Pilot study	EbM course	Live	142	Self-help groups, patients and representatives participants: patients, patient counsellors, consumer representatives, health professionals	N/A
I-14	Hinneburg *et al*. 2020	Germany	Pilot study	EbM course	Live and online, asynchronous	29	Physicians, medical students Participants: physicians and health professionals	Medical association
I-15	Rahner *et al*. 2022	Germany	Pilot study	EbM course	Live	8	Teachers for health professions	Vocational school for health professions
I-16	Zacher *et al*. 2022	Germany	Pilot study	Web tool for risk communication	Online, asynchronous	22	Public Participants: lay people, health professionals	Internet
I-17	Muscat *et al*. 2015	Australia	Evaluation and pilot study	Shared decision-making training	Live	26	People with limited HL	Adult education provider
	Muscat *et al*. 2017a	Australia	Interview study	Shared decision-making training	Live	22	People with limited HL	Adult education provider
	Muscat *et al*. 2017b	Australia	Interview study	Shared decision-making training	Live	11 teachers	People with limited HL	Adult education provider
	Muscat *et al*. 2019	Australia	cRCT	Shared decision-making training	Live	308	People with limited HL	Adult education provider
	McCaffery *et al*. 2019	Australia	cRCT	Shared decision-making training	Live	308	People with limited HL	Adult education provider
I-18	Muscat *et al*. 2021	Australia	Case study	SUCCESS app (supporting people with chronic kidney disease in shared decision-making)	Online, asynchronous	N/A	Patients with chronic kidney disease	Internet
I-19	Serbim *et al*. 2020	Brazil	Quasi-experimental study	Alpha Health Program (CHL course)	Live	42	Older adults	Primary care
I-20	Scull *et al*. 2018	USA	cRCT	Media Aware: sexual health programme for young adults	Online, asynchronous	184	University students	College
I-21	Aghazadeh *et al*. 2020	USA	Pilot study	HL programme	Live	365 students 5 teachers	Students	Primary school
I-22	Smart *et al*. 2016	USA	Pilot study	Information Literacy Workshop	Live	20	Students of nursing	Higher education
I-23	Aspinall *et al*. 2012	USA	Pilot study	HL workshop	Live	63	Older adults	Residential aged care facility
I-24	Bauquier *et al*. 2024	France	Case study	Patient–researcher training	Live	11	People living with or with a history of cancer	Higher education
I-25	Bay *et al*. 2017	New Zealand	Mixed-methods study	School–university partnership programme	Live	210	Students	Primary school
I-26	Berr *et al*. 2021	USA	Case study	Remote summer programmes for Students	Online, synchronous and asynchronous	21	University students	Internet
I-27	Bloss *et al*. 2022	USA	Case study	Information literacy training	Online, asynchronous	3 FG	Health-related social workers	Internet
I-28	Chang *et al*. 2025	Taiwan	Pilot-RCT	Virtual reality-based dementia prevention programme	Online, asynchronous	60	Older adults with mild cognitive impairment	Nursing homes, day care centres, community centres
I-29	Chang and Chen 2023	Taiwan	Quasi-experimental mixed-method-study	Web-based drug prevention programme	Online, asynchronous	1065	Adults	Internet
I-30	Chen 2019	China	Mixed-method-study (dissertation)	WeChat app to improve HL	Online, synchronous	389 8 FG	Mothers	Internet
I-31	Cobban and Seale, 2003	Canada	Evaluation study	Course to improve information literacy	Live	40	University students	Higher education
I-32	Goodman *et al*. 2010	USA	Evaluation study	CARES-Fellow-Training (Public health research training)	Live	19	Minority groups and medically underserved populations	Library
	Goodman *et al*. 2012	USA	Evaluation study	CARES-Fellow-Training (Public health research training)	Live	19	Minority groups and medically underserved populations	Library
	Coats *et al*. 2015	USA	Mixed-method-study	CARES-Fellow-Training (Public health research training)	Live	44	Minority groups and medically underserved populations	Higher education
I-33	Earl *et al*. 2019	USA	Case study	HL module	Live	N/A	Pharmacy Students	Higher education
I-34	Fiordelli *et al*. 2023	Switzerland	Pilot study	Training course on CHL and scientific literacy	Live	97	Students	Secondary school
I-35	Greenberg and Wang, 2012	USA	Case study	Online health videos for public and school-based health education	Online, asynchronous	20	Students	Secondary school
I-36	Keselman *et al*. 2015	USA	Interview study	Youth programme for health leadership and responsibility	Live	10	Underprivileged students	Secondary school
I-37	Keselman *et al*. 2019	USA	Case study	Health information, leadership and empowerment programme	Live	78	University students	Adult education provider
I-38	König *et al*. 2022	Germany	Evaluation study	E-learning course to improve (digital) HL	Online, asynchronous	323	Students	Secondary school
I-39	Lin *et al*. 2021	Taiwan	Quasi-experimental study	Drug use prevention course	Live	648	Students	Secondary school
I-40	Nazri 2019	Malaysia	Editorial case study	Workshop on scientific literacy and HL	Live	106	Students	Secondary school
I-41	Peralta *et al*. 2022	Australia	Mixed-method-case study	(Critical) HL training course	Live and online, synchronous	9	Teachers	Secondary school
	Peralta *et al*. 2025	Australia	Case study	(Critical) HL training course	Live and online, synchronous	3	Teachers	Secondary school
I-42	Roux *et al*. 2023	Australia	Quasi-experimental mixed-method-study	My Vital Cycles®, ovulatory menstrual health programme	Live and online, asynchronous	94	Female students	Secondary school
I-43	Stassen *et al*. 2020	Germany	cRCT	Web-based programme to improve HL	Online, asynchronous	532	Students	Vocational school
I-44	Sykes and Wills 2018	U.K.	Case study	CHL community learning programme	Live	24	Parents	Community
I-45	Vamos *et al*. 2012	USA	Case study	‘Women's Health’ undergraduate course	Live	N/A	Female university students	Higher education
I-46	van Moorsel 2001	USA	Case study	Mini-Medi-School	Live	N/A	Public	Higher education
I-47	Weng *et al*. 2025	Taiwan	Quasi-experimental study	Interactive digital drug prevention programme	Online, asynchronous	168	Students	Internet
I-48	Dixon *et al*. 2023	New Zealand	Interview study	Health education curriculum	Live	25	Students	Secondary school
I-49	Milne *et al*. 1996	U.K.	Case study	Consumer health workshop	Live	84	Health counsellors	Workplace
I-50	Murray *et al*. 2020	USA	Case study	Training course for peer navigators	Live	N/A	Peer navigators	Community
I-51	Smith *et al*. 2019	Australia	RCT	Web or DVD plus booklet to improve HL and decisional certainty	Online, asynchronous	153	Adults aged >65 years	Internet
I-52	Tsai *et al*. 2018	Taiwan	Quasi-experimental study	HL programme	Live	223	Immigrants	Community
I-53	Gould *et al*. 2010	USA	Case study	Just Health Action, programme to promote health equity	Live	—	Students	Secondary school
	Mogford *et al*. 2011	USA	Case study	Just Health Action, programme to promote health equity	Live	—	Students	Secondary school

*n* = number of participants; FG = focus groups; SDM = shared decision-making; IHC = informed health choices; N/A = not applicable/not available; cRCT = cluster randomized controlled trial; RCT = randomized controlled trial

The included interventions span 29 years (1996–2025), with a marked increase in first publications from 2016 onwards. Using the first publication per intervention for temporal classification, most interventions were first published between 2021 and 2025 (*n* = 21) and between 2016 and 2020 (*n* = 18).

Case study designs were most common (*n* = 16), followed by pilot studies (*n* = 11), mixed-methods studies (*n* = 8) and development studies of interventions (*n* = 5). Experimental designs included cluster-randomized trials (*n* = 5), randomized controlled trials (*n* = 3) and quasi-experimental studies (*n* = 5). Follow-up assessments were reported for three interventions and process evaluations were identified for three interventions.

### Target groups

Most interventions targeted school students (*n* = 19; I-01, I-03–I-06, I-11, I-21, I-25, I-34–I-36, I-38–I-40, I-42, I-43, I-47–I-48, I-53) and, partly in parallel, teachers (*n* = 8; I-01, I-03–I-06, I-15, I-32, I-41). Parents or caregivers were addressed in two school-linked formats (I-02, I-10) and one community-based learning offer (I-44). In higher education, university students were a common target group (*n* = 9; I-07, I-08, I-14, I-20, I-26, I-31, I-33, I-37, I-45).

Five interventions targeted specific professional groups (I-12, I-14, I-15, I-22, I-27), including diabetes educators, physicians and medical students, teachers for health professions, nursing students and health-related social workers. Fourteen interventions targeted patient or public groups outside institutional education, including people with low HL (I-17), chronic kidney disease patients (I-18), cancer survivors (I-24), older adults (I-19, I-23, I-28, I-51), mothers (I-30), peer navigators (I-50), migrants (I-52), minority or medically underserved populations (I-32) and general public audiences (I-16, I-29, I-46).

Overall, seven interventions explicitly addressed mixed target groups (*n* = 7; I-01, I-09, I-10, I-13, I-14, I-16, I-26), including combinations of school or university students and teachers, public and caregiver audiences and multi-interest-holder formats involving patients or consumer representatives together with health professionals.

### Settings

As interventions could be assigned to more than one setting, the categories reported below are not mutually exclusive. Interventions were most frequently implemented in formal education settings, particularly primary and secondary schools (*n* = 19; I-01–I-06, I-11, I-21, I-25, I-34–I-36, I-38–I-42, I-48, I-53) and universities or colleges (*n* = 12; I-07, I-09, I-13, I-20, I-22, I-24, I-26, I-31–I-33, I-45–I-46). Internet-based settings were likewise frequent (*n* = 12; I-06, I-08, I-10, I-16, I-18, I-20, I-26–I-27, I-29–I-30, I-47, I-51). Less common settings comprised vocational schools (*n* = 2; I-15, I-43), community-based settings (*n* = 6; I-17, I-28, I-37, I-44, I-50, I-52) and healthcare or care contexts (*n* = 6; I-12, I-14, I-19, I-23, I-28, I-49).

RQ1a: Which theoretical underpinnings and frameworks informed intervention development?

Overall, 51 interventions referred to at least one theoretical, conceptual or model-based foundation, while only a small number of interventions (*n* = 2; I-26, I-45) did not report any identifiable theoretical underpinning.

### HL models and conceptualizations of CHL

Nutbeam’s (2000) model of functional, interactive and critical HL was the most frequently cited framework (*n* = 14; I-17, I-18, I-28, I-30, I-34, I-39, I-41, I-42, I-44, I-47, I-48, I-51, I-52, I-53).

Less frequently, interventions referred to the integrated model of HL by Sørensen *et al*. (2012) (*n* = 2; I-38, I-52), the Institute of Medicine framework (2004) (*n* = 1; I-33) or specific conceptualizations of CHL (Benkert and Abel 2023) (*n* = 1; I-41).

In addition, 10 interventions referred to HL in general without specifying an underlying model (*n* = 10; I-10, I-11, I-13, I-14, I-15, I-21, I-24, I-25, I-43, I-50).

Taken together, these findings suggest that many interventions were informed by concepts overlapping with CHL, while explicit use of CHL frameworks remained limited.

### CHL-related concepts

Evidence-based medicine (EbM) was the most frequently cited framework for critical appraisal-related competencies (*n* = 15; I-07, I-12, I-14, I-15, I-20, I-22, I-24, I-27, I-31, I-32, I-33, I-41, I-49, I-50, I-51), typically referring to established steps such as formulating questions, searching for evidence, critically appraising information and applying findings to decision-making (Sacket *et al.* 1996).

Scientific literacy frameworks (OECD 2017) were also commonly applied (*n* = 5; I-07, I-08, I-16, I-20, I-37), alongside critical thinking frameworks (OECD 2024) in education (*n* = 4; I-08, I-16, I-20, I-41).

In addition, several interventions drew on related approaches to critical appraisal, including specific appraisal frameworks or tools (*n* = 6; I-07, I-08, I-20, I-33, I-41, I-47) as well as concepts of risk and statistical literacy (*n* = 4; I-13, I-16, I-33, I-47).

### Behavioural and implementation-related frameworks

Behavioural theories were applied in a small subset of interventions, most commonly Social Cognitive Theory (Bandura 1986) (*n* = 2; I-10, I-39), the Theory of Planned Behaviour (Ajzen 1991) (*n* = 3; I-16, I-29, I-42) and the Health Belief Model (Rosenstock *et al*. 1988) (*n* = 1; I-52). These were often complemented using specific constructs such as self-efficacy (Bandura 1977) (*n* = 3; I-10, I-39, I-52).

### Methodological development approaches and frameworks

Iterative human-centred design approaches were most prominently applied within the Informed Health Choices (IHC) (Chalmers *et al*. 2018) interventions (*n* = 6; I-01, I-02, I-03, I-04, I-05, I-06), characterized by repeated rounds of prototyping, user testing and refinement.

Structured intervention development frameworks, most notably the Medical Research Council (MRC) framework for complex interventions (Skivington *et al*. 2021), were explicitly referenced in a smaller number of interventions (*n* = 5; I-10, I-14, I-16, I-29, I-42). In addition, one intervention applied the ADDIE instructional design model (*n* = 1; I-16).

Participatory or co-design approaches were reported in twelve interventions (*n* = 12; I-01, I-03, I-05, I-06, I-17, I-27, I-29, I-35, I-41, I-43, I-49, I-52), involving interest-holders such as target groups, teachers or patients through qualitative methods or advisory structures.

Adaptation or transfer of existing programmes or curricula was described in 17 interventions (*n* = 17; I-02, I-03, I-04, I-05, I-11, I-13, I-15, I-20, I-23, I-27, I-30, I-32, I-44, I-45, I-48, I-51, I-53).

RQ1b: Which educational goals are employed in these interventions?

Most interventions aimed to strengthen the ability to locate evidence-based health information and to appraise information sources, e.g. by teaching search strategies, evaluation of information sources and use of reliable information resources (*n* = 30; I-02, I-05, I-06, I-07, I-08, I-10, I-11, I-13, I-14, I-15, I-20, I-24, I-26, I-27, I-30, I-31, I-32, I-33, I-35, I-36, I-38, I-40, I-41, I-44, I-46, I-48–I-51, I-53). Many interventions also addressed elements of informed and shared decision-making, such as asking questions, weighing benefits and harm, incorporating values and preferences, and structuring decisions (*n* = 29; I-01, I-02, I-03, I-05, I-06, I-09, I-10, I-11, I-13, I-14, I-15, I-17, I-18, I-19, I-20, I-21, I-22, I-23, I-28, I-29, I-30, I-39, I-41, I-47, I-48, I-50, I-51, I-52, I-53). A third common focus was improving understanding of research, science and evidence, including study design basics, bias, interpreting results and the role of systematic reviews (*n* = 22; I-02, I-04, I-05, I-06, I-07, I-08, I-10, I-11, I-14, I-15, I-24, I-26, I-30, I-32, I-40, I-41, I-44, I-46, I-49, I-50, I-51, I-53). Critical appraisal of health information was explicitly named as a goal in 20 interventions, e.g. recognizing unreliable claims, judging the evidence base and weighing benefits and harm of prevention or treatment options (*n* = 20; I-01, I-02, I-04, I-05, I-07, I-08, I-10, I-14, I-15, I-17, I-28, I-29, I-30, I-33, I-38, I-39, I-47, I-49, I-51, I-36). Less frequently, interventions targeted statistical and risk competencies, such as understanding probabilities, absolute versus relative risks and interpreting effect estimates (*n* = 10; I-13, I-16, I-17, I-20, I-25, I-33, I-36, I-39, I-47, I-50). Additional goals related to media and communication literacy, including reflection on media messages and media-related influences on decisions (*n* = 9; I-02, I-05, I-07, I-08, I-13, I-20, I-21, I-30, I-47), and to health rights, social participation or the social determinants of health (*n* = 8; I-10, I-17, I-19, I-32, I-37, I-44, I-52, I-53).

RQ1c: Which educational and implementation strategies are used for CHL interventions?

### Integration into existing curricula vs. stand-alone interventions

Interventions were most commonly embedded in teaching curricula as structured units, teaching materials or modules (*n* = 18; I-01, I-03, I-04, I-05, I-07, I-11, I-21, I-25, I-30, I-34, I-36, I-37, I-38, I-41, I-42, I-44, I-48, I-53). Stand-alone interventions were widely used as workshops, training sessions and short courses (*n* = 28; I-01, I-03–I-05, I-07, I-11–I-17, I-21–I-24, I-31–I-32, I-34, I-38, I-39, I-40, I-42, I-45, I-47, I-49, I-50, I-52, I-53). Where specified, these formats were commonly delivered as multi-session or modular programmes with sequential units (*n* = 10; I-07, I-14–I-15, I-17, I-24–I-25, I-32–I-33, I-38, I-40).

### Delivery modalities

Regarding modality, delivery was coded as in-person (face-to-face), online (web-based/online) or blended learning (combining face-to-face and online components). Across all interventions, delivery most often occurred in-person (*n* = 29; I-01, I-04, I-11, I-12, I-13, I-15, I-17, I-19, I-21, I-22, I-23, I-24, I-25, I-28, I-32, I-33, I-34, I-36, I-37, I-39, I-40, I-44, I-45, I-46, I-48, I-49, I-50, I-52, I-53), followed by online-only formats (*n* = 17; I-06, I-08, I-09, I-10, I-16, I-18, I-20, I-26, I-27, I-29, I-30, I-35, I-38, I-43, I-47, I-49, I-51) and blended learning approaches (*n* = 7; I-03, I-05, I-07, I-14, I-31, I-41, I-42). One intervention relied on a device-based audio format delivered via a portable media player (I-02).

For interventions with an online component (online-only and blended; *n* = 24; I-03, I-05, I-06, I-07, I-08, I-09, I-10, I-14, I-16, I-18, I-20, I-26, I-27, I-29, I-30, I-31, I-35, I-38, I-41, I-42, I-43, I-47, I-48, I-51), delivery was predominantly online-asynchronous (*n* = 20; I-05, I-06, I-07, I-08, I-09, I-10, I-14, I-16, I-18, I-20, I-27, I-29, I-31, I-35, I-38, I-41, I-42, I-43, I-47, I-51). Online-synchronous delivery was rare (I-03, I-30) and one intervention explicitly combined synchronous and asynchronous elements (I-26). In addition, one intervention used an immersive virtual-reality headset-based learning environment (I-28), implemented in a face-to-face setting with facilitated reflection.

### Teaching methods and didactic concepts

Across interventions, teaching methods most commonly comprised exercises, facilitated discussions and guided learning activities (*n* = 32; I-03, I-04, I-07, I-10–I-15, I-18, I-19, I-22–I-25, I-27, I-28, I-31–I-33, I-35, I-37, I-39, I-41–I-44, I-47, I-49–I-51, I-53). Podcasts, explanations via videos and animations were reported in nine interventions (*n* = 9; I-02, I-06, I-19, I-27, I-29, I-35, I-45, I-47, I-48), whereas game- and simulation-based approaches were comparatively rare (*n* = 5; I-08, I-19, I-28, I-39, I-47). Learning was commonly organized through a combination of group-based activities (e.g. work and discussions in small groups; *n* = 31; I-01–I-05, I-07, I-11, I-15, I-17, I-19–I-23, I-30, I-32, I-34, I-35, I-37, I-39, I-41–I-42, I-44–I-47, I-49–I-53) and individual work or self-study phases (*n* = 15; I-05–I-08, I-14, I-29, I-32–I-33, I-37, I-43–I-45, I-50, I-51, I-53).

Role-play and scenario-based practice activities were used in five interventions (*n* = 5; I-05, I-33, I-39, I-47, I-50). Case- or problem-based learning approaches were reported in three interventions (*n* = 3; I-15, I-16, I-34), while flipped-classroom elements were described in two interventions (*n* = 2; I-07, I-34).

### Duration

Intervention duration varied widely, ranging from micro-learning formats to multi-month programmes. Micro-learning modules are very brief, self-contained learning units that typically focus on a single key concept or skill and can often be completed within a few minutes, although no universally agreed duration threshold exists (e.g. short podcast episodes, I-02 or a self-guided online tool on risk reduction I-16). Several interventions reported clearly defined multi-week or longer delivery schedules, including semester-based implementations or sequences over several weeks (*n* = 6; I-05, I-17, I-21, I-39, I-42, I-53), for example curriculum-based delivery during a school term (I-05) or structured longer-running programme implementation (I-42).

RQ1d: Which dimensions of CHL are addressed by these interventions?

Following Benkert and Abel (2023), CHL was conceptualized across three core components: critical appraisal of health information, understanding of social determinants of health, and individual and collective action. Interventions were first classified by the CHL dimensions they addressed. Most interventions focused on the critical evaluation dimension (*n* = 51) and addressed, for example, the critical evaluation of health claims, evidence and misinformation (e.g. I-11). This was followed by the individual and collective action dimension (*n* = 42), with interventions aiming to strengthen informed decision-making, participation and empowerment in health-related issues in everyday life and society (e.g. I-17 and I-48). Explicit attention to social determinants of health was comparatively rare (*n* = 5) and focused on understanding how social, economic and environmental conditions influence health (e.g. I-44 and I-53) whereas interventions addressing individual and collective action aimed to strengthen informed decision-making, participation and empowerment with health-related issues in everyday life and society (e.g. I-17 and I-48). Secondly, we examined how many CHL dimensions each intervention covered. Most interventions addressed two dimensions (*n* = 35), typically combining critical appraisal with action/decision-making. Thirteen interventions focused on a single dimension (predominantly appraisal; two interventions addressed action/decision-making only: I-21 and I-23) and five interventions covered all three dimensions (*n* = 5; I-03, I-37, I-44, I-48, I-53).

RQ1e: To what extent have the interventions been evaluated and what approaches or measures have been used?

Evaluation approaches commonly combined summative outcome assessment with formative or process-oriented components designed to identify usability and context requirements and to inform adaptation. Quantitative questionnaire-based assessments were most frequently reported (*n* = 18; I-01, I-04–I-05, I-09–I-11, I-13–I-15, I-18, I-20, I-24, I-31–I-33, I-34, I-41, I-52), often complemented by qualitative interviews or focus groups (*n* = 19; I-02–I-03, I-05, I-07, I-09–I-10, I-12–I-13, I-15, I-16, I-18, I-20, I-24, I-32–I-33, I-34, I-37, I-42, I-49, I-52). Observational approaches, such as structured session or classroom observations, were reported for seven interventions (I-01, I-03, I-05, I-15, I-21, I-39, I-42). Monitoring procedures were described in isolated cases (I-01, I-24). Regarding evaluation outcomes, assessments most frequently addressed acceptability, comprehensibility, feasibility, satisfaction (*n* = 8; I-08, I-10, I-16, I-20, I-24, I-32, I-34, I-49), fidelity and implementation aspects (*n* = 8; I-01–I-02, I-08, I-13, I-24, I-27, I-52, I-53) and prototype testing within development (*n* = 5; I-01, I-05, I-08–I-10). Based on formative findings, documented adaptations were reported for eight interventions (I-01–I-05, I-14, I-16, I-17).

Measurement instruments were explicitly reported for 25 interventions, comprising 21 distinct measurement approaches. Within the IHC interventions, the claim evaluation tools were those used most consistently (Austvoll-Dahlgren *et al*. 2017). In the other studies, outcomes were assessed using a mix of validated instruments and intervention-specific tools, such as the Critical Health Competence test (Steckelberg *et al*. 2009a, 2009b) and an EbM competence test (Berger *et al*. 2010). Broader HL instruments were used in some interventions, for example the HL questionnaire (Osborne *et al*. 2013) and the newest vital sign (Weiss *et al*. 2005), alongside study-specific questionnaires aligned with targeted outcomes.

Economic evaluation was rarely reported. Only two interventions provided explicit cost or resource information (I-01, I-06), including a quantified school-level estimate for one IHC programme.

RQ2: Which barriers and facilitators related to promoting CHL are reported?

Facilitators were reported for 19 of 53 interventions (I-01–I-05, I-07, I-09, I-14–I-17, I-21, I-24, I-31–I-33, I-34, I-41, I-42) and barriers for 21 of 53 interventions (I-01–I-05, I-07, I-09, I-11, I-13–I-17, I-21, I-24, I-31, I-32, I-34, I-41, I-42, I-53). Across these studies, we identified nine recurring facilitators and eight recurring barriers related to promoting and implementing CHL interventions. Facilitators most frequently reported were structured materials, preparation and support of deliverers, participatory development, contextual fit, interactive formats and digital/multi-media elements, whereas barriers frequently related to time and workload, technical hurdles, group-management challenges, language and literacy demands, organizational constraints, limited motivation, pandemic-related disruptions and insufficient clarity or tailoring of content. Table 2 provides a detailed overview of all facilitators and barriers, including rate of recurrence, intervention IDs, and illustrative examples.

**Table 2:** Barriers and facilitators for implementing CHL interventions.

Main facilitator *(n; I-IDs)*	Illustrative examples
Structured, ready-to-use materials *(n* = *9; I-01, I-03–I-05, I-14–I-17, I-42)*	*I-03:* Students/teachers highlighted the comic and exercise book with realistic examples and engaging activities. *I-15:* Facilitators included supplementary course materials and provision of work materials. *I-1*7: Teachers described the programme as very clear and well structured, and learners valued the pocket card as a practical resource for future reference.
Training/coaching and support for deliverers *(n* = *9; I-01, I-03–I-05, I-14–I-15, I-17, I-21, I-42)*	*I-01:* Support from school authorities and colleagues helped secure adequate time and permission to teach the lessons. *I-15:* Qualified teachers and ‘time off for the training’ were described as conducive to implementation. *I-17:* Deliverers reported that structured preparation and support enabled delivery for learners with limited literacy.
Participatory or user-centred development *(n* = *7; I-01–I-03, I-05, I-09, I-16, I-17)*	*I-02:* User testing informed iterative adaptations of the intervention materials, including the selection of preferred language versions and improvements to structure and sequencing. *I-16:* Using an everyday-life vignette to introduce the problem increased participants’ curiosity and motivation by making the topic immediately relatable and noticeable. *I-17:* Pre-implementation qualitative interviews with students closely matching the target group generated insights into relevant beliefs and expectations, which were used to tailor the shared decision-making programme.
Contextual fit and relevance *(n* = *8; I-01, I-03–I-05, I-15, I-21, I-24, I-42)*	*I-05:* Teachers and students experienced the lessons as relevant for everyday life and other subjects. *I-24:* High contextual relevance to participants’ lived experience supported involvement in the programme. *I-42:* Participants emphasized that presenting potentially complex science through realistic, relatable scenarios increased identification with the content and supported perceived usefulness.
Interactive/activating pedagogy *(n* = *11; I-01–I-02, I-05, I-07, I-14–I-15, I-17, I-32–I-34, I-41)*	*I-01:* Group activities and discussions were described as attractive for learners. *I-14:* Interactive instructional design was appreciated because it promoted exchange and discussions. *I-32:* Participants reported that small-group activities were helpful and beneficial, and they rated the interactive session format as supportive for involvement and learning.
Digital/multi-media elements improving access/clarity *(n* = *8; I-02, I-07, I-14, I-16, I-31, I-34, I-41, I-42)*	*I-02:* Podcast delivery and language options supported accessibility and understanding. *I-08:* A serious-game format was perceived as attractive and helped students practise critical thinking about health claims by actively evaluating the evidence behind the claims. *I-41:* Digital resources complemented live delivery and supported access to teaching content.
Organizational support and enabling conditions *(n* = *6; I-01, I-03–I-05, I-21, I-42)*	*I-01:* Leadership support enabled protected time and implementation conditions for teachers. *I-05:* Teachers reported that delivering the lessons in ‘projector mode’ provided a practical framework and made classroom implementation more feasible. *I-42:* Organizational support helped integrate programme components and peer-based teaching.
Motivation and perceived usefulness *(n* = *8; I-01–I-02, I-05, I-07, I-17, I-24, I-32, I-42)*	*I-01:* The children enjoyed the lessons and looked forward to them, reflecting positive attitudes towards the materials. *I-32:* Participants reported satisfaction with programme logistics/structure and willingness to continue. *I-42:* Participants reported that the information was useful and helpful and that it increased their comfort and confidence over time.
Cooperation and networks *(n* = *7; I-01, I-04–I-05, I-14, I-32, I-41–I-42)*	*I-01:* Support from school leadership and colleagues facilitated implementation by enabling scheduling within the timetable, allocating time for preparation and endorsing participation. *I-32:* Fellows reported willingness to collaborate with peers on pilot projects. *I-42*: Including medical students as peer educators was perceived as particularly valuable; many participants described the peer-based teaching component as one of the programme’s key strengths.

Main barrier *(n; I-IDs)*	Illustrative examples
Time constraints and workload *(n* = *7; I-01–I-05, I-13, I-42)*	*I-01:* Teachers reported that limited lesson time and competing curricular demands constrained delivery, leaving insufficient time to cover all key concepts and activities as intended. *I-04:* Teachers described time constraints within the school day/curriculum. *I-13:* Participants reported time constraints and limited opportunities for follow-up exchange/support.
Technical and digital hurdles *(n* = *11; I-01–I-03, I-05, I-07, I-14–I-17, I-31, I-41)*	*I-03:* Remote sessions faced technical issues and reduced interaction. *I-07:* Students reported usability and navigation problems within the learning management system and they suggested clearer guidance for using the platform. *I-14:* The learning management system caused some technical problems.
Teaching and group-management challenges *(n* = *12; I-01–I-05, I-07, I-11, I-14–I-17, I-32)*	*I-05:* Teachers found class discussions hard to organize when students used computers. *I-09:* Facilitators reported challenges in moderating discussions and sustaining active participation in larger or heterogeneous groups. *I-32:* Managing group processes and participation posed challenges during training sessions.
High language and literacy demands *(n* = *8; I-05, I-07, I-09, I-11, I-13, I-15, I-17, I-41)*	*I-09:* Terminology/readability required refinement to improve comprehension. *I-11:* Differing proficiency levels meant original English-language articles were not feasible for most students. *I-13:* Reported barriers included difficulty reading English-language publications, limited access to databases and full-text articles, organizational constraints and time pressure.
Organizational and structural constraints *(n* = *12; I-01–I-05, I-07, I-09, I-13–I-14, I-21, I-41, I-53)*	*I-05:* Curriculum and timetable constraints limited feasibility; schools lacked time to teach all lessons. *I-14:* Organizational barriers (e.g. limited institutional embedding) constrained implementation conditions. *I-53:* Structural school conditions influenced what could realistically be delivered.
Low motivation or initial reluctance *(n* = *7; I-02–I-05, I-07, I-17, I-53)*	*I-05*: Resistance to certain delivery modes led to adaptations (e.g. dropping student-computer mode). *I-21:* Teachers noted that varying motivation and buy-in across classes influenced uptake and required additional efforts to encourage participation. *I-42:* Some participants reported initial hesitancy to engage, improving only over time.
Pandemic-related disruptions *(n* = *3; I-03, I-41, I-42)*	*I-03:* COVID-19 restrictions caused absences and intermittent remote learning. *I-41:* Planned follow-up professional development sessions were not possible due to lockdowns. *I-42:* Programme delivery and engagement were affected by pandemic conditions and restrictions.
Insufficient clarity or tailoring of content *(n* = *8; I-01–I-02, I-05, I-09, I-14, I-17, I-24, I-34)*	*I-02:* Some listeners confused claims and key messages, indicating need for clearer explanations. *I-14:* Feedback indicated that the content was initially too abstract for some participants; adding a structured introductory input with practical examples was necessary to improve comprehension and applicability. *I-17:* Early iterations lacked a clear, contextualized explanation of shared decision-making and how to use the core questions in real consultations.

*n* = number of interventions; I-ID = intervention identification number.

Within IHC-related interventions, an additional barrier concerned tensions between intervention messages and participants’ prior beliefs or experiences, which could trigger scepticism or resistance. IHC interventions also repeatedly reported that understanding and teaching the underlying key concepts was perceived as demanding by learners and or teachers (*n* = 4; I-01, I-03–I-05).

## Discussion

This scoping review mapped 53 distinct interventions (in 81 publications) that aimed to promote CHL. Interventions were implemented mostly in educational settings and primarily targeted critical appraisal of health information and individual action, such as informed decision-making. In contrast, explicit attention to social determinants of health and collective action was rare. Delivery relied typically on curriculum-embedded units or modular trainings, increasingly supplemented by digital elements. Reported barriers and facilitators highlighted the finding that implementation is strongly shaped by practical conditions such as time, resources, technical infrastructure and the preparation and support of those delivering the intervention.

The rise in interventions may reflect the growing conceptual differentiation of CHL from 2010 on. At the same time, some earlier interventions predate contemporary CHL definitions and align more closely with adjacent constructs, such as EbM-related competence gains (Milne and Oliver 1996), making classification within narrowly defined CHL less straightforward.

For the IHC interventions, development was consistently described as iterative and person-centred, including prototyping, repeated testing and revision and was supported by dedicated development publications (*n* = 6; I-01–I-06). In comparison, many other interventions were not newly developed but rather were adaptations or extensions of existing curricula or formats (*n* = 17; I-02–I-05, I-11, I-13, I-15, I-20, I-23, I-27, I-30, I-32, I-44, I-45, I-48, I-51, I-53). However, modifications, their rationales and contextualization were often only rudimentarily described. For five interventions development information was not extractable (I-24, I-26, I-36, I-40, I-50). These deficits in reporting hinder replication transfer to new contexts and realistic assessment of implementation potential. However, the detailed reporting of development processes within the IHC programme should be interpreted in light of its extensive publication record. For many other interventions, only a single publication was available. Consequently, the greater level of detail available for the IHC programme may partly reflect differences in publication coverage rather than differences in intervention development itself. Moreover, because the IHC programme represents a substantial proportion of the available evidence on intervention development, conclusions regarding the value of iterative, person-centred development should be interpreted with caution and cannot necessarily be generalized to the broader CHL intervention literature. These patterns point to a broader methodological issue that is central to the MRC framework (Skivington *et al*. 2021): intervention identification and development should be treated as explicit, reportable stages that underpin later feasibility testing, evaluation and implementation. However, evidence from other fields suggests that references to MRC guidance have increased over time, while reporting quality remains heterogeneous (Goodwin *et al*. 2019). In health promotion research in general, deficits in intervention development are increasingly highlighted as a central challenge for methodological quality (Dichter 2022). A key reason is the lack of explicit programme theory. Logic models and theories of change can make assumptions about mechanisms and context interactions explicit, thereby strengthening adaptation, implementation and evaluation (Skivington *et al*. 2021, Dichter 2022). In CHL interventions, this appears particularly relevant because many programmes operate at the interface of education, health communication and health systems, where contextual demands are complex and variable. Without transparent programme theory, however, it often remains unclear which intervention components drive effects, how outcomes are expected to emerge and which elements are transferable across settings, thereby limiting the interpretability, adaptation and scalability of CHL interventions.

Notwithstanding these shortcomings, it is encouraging that a relevant share of interventions (*n* = 13) explicitly incorporated target-group perspectives during development, for example via focus groups, interviews, advisory boards or co-design elements. This is broadly consistent with MRC recommendations to involve relevant interest-holders across phases (Skivington *et al*. 2021). Yet the depth of participation and its influence on concrete design decisions often remained unclear, limiting conclusions about how needs-based and context-specific interventions ultimately were.

A further interpretive point concerns the frequent implicitness of CHL framing. In most interventions, the CHL link was inferred from aims and content rather than explicitly labelled as CHL. This reflects a recurrent problem in the literature. De Wit *et al*. (2017) and Romanova *et al*. (2024) reported that the term ‘critical health literacy’ yielded no database records when combined with additional search terms and therefore could not be used as a search concept, requiring CHL to be assessed via inclusion criteria and often identified implicitly in included interventions. Our findings are consistent with this pattern.

Content-wise, interventions were heavily concentrated on an ‘appraisal-centred’ interpretation of CHL, which corresponds to ongoing criticism that CHL is often reduced to an individual cognitive performance of higher order (Chinn 2011, Sykes *et al*. 2013, 2025). Within the included articles, appraisal-oriented and individual decision-making interventions predominated, reflecting a strong influence of EbM, scientific literacy and critical thinking. These competencies are central to CHL. The identified lower proportion of interventions that take social determinants and collective action into account may be attributable to various reasons. On the one hand, this might be because the search strategy was primarily oriented towards interventions related to health information appraisal and various synonymous and related concepts were included in the search strategy, whereas no explicit search terms for social determinants and collective action were used. This may also suggest that these dimensions are not consistently associated with CHL, otherwise, they would have been identified. On the other hand, collective action and social determinants may remain less developed in intervention practice. This raises broader questions about whether CHL promotion is framed mainly as strengthening individuals or also as shaping enabling environments.

This narrowing down has implications beyond conceptual clarity. If CHL is framed primarily as an individual responsibility for ‘correct’ decisions, a normative shift of accountability to individuals becomes plausible (Sykes *et al*. 2013). In disadvantaged living conditions, translation of competence into action may remain constrained despite improved knowledge or attitudes. Accordingly, reducing CHL primarily to individual competencies may narrow attention to personal decision-making capacities, while underestimating the influence of institutional arrangements, social conditions and information environments on health-related practices. This stands in tension with health promotion-oriented conceptualizations of CHL, which emphasize empowerment, social determinants of health and collective action. Moreover, considering established barriers to EbM implementation (Halalau *et al*. 2021, Ehrenbrusthoff *et al*. 2022, Wang *et al*. 2023b), a further tension may arise: CHL competencies may be constrained in clinical contexts where evidence-based practices are applied inconsistently.

The diversity of outcome measures identified in this review reflects a broader challenge within the field: While several instruments have been developed to capture specific dimensions of CHL, no consensus exists regarding a comprehensive measure of the construct (Benkert and Abel 2023). Moreover, Chinn (2011) has argued that CHL encompasses context-dependent cognitive, social and political dimensions which may not be adequately captured by standardized measurement alone. As a result, evaluation approaches often emphasize particular components of CHL, most commonly appraisal-related competencies, while broader dimensions may remain under-represented.

Finally, our findings on educational implementation align with educational research on critical thinking. CHL aims were most often realized through structured curricular or course formats using activating methods such as guided exercises and discussion, occasionally supplemented by problem-oriented or simulation elements. Prior syntheses similarly emphasize the value of structured instructional sequences that integrate active learning, practice and feedback for supporting critical thinking (Oxman *et al*. 2024, Prokop-Dorner *et al*. 2024). In addition, the most common barriers and facilitators in our review, especially time and resource constraints and the importance of training and support for deliverers, are consistent with implementation research on school programmes, which emphasizes leadership prioritization, protected time and supportive staff structures as pre-requisites for sustained quality of delivery (Herlitz *et al*. 2020, Ponsford *et al*. 2022, Prenger *et al*. 2022, Ulla and Poom-Valickis 2023).

Economic evaluation was rarely reported, which corresponds to the generally limited health-economic evidence for (C)HL interventions. While some international analyses suggest that school-based prevention programmes can yield positive economic effects under certain conditions (Stielke *et al*. 2019, Ekwaru *et al*. 2021), CHL-specific cost-effectiveness evidence remains scarce.

Overall, the interventions included in this review reflect a predominantly individualized and appraisal-centred operationalization of CHL. However, there is an urgent need for a clear operationalization of the concept of CHL.

### Implications

Because most interventions were implemented in school settings, schools appear to be a central lever for promoting CHL early, with low threshold and population-wide reach. A key advantage is that existing educational infrastructures can be used, which may reduce implementation burden when compared with newly established services. However, scalability depends on context fit and transferability. However, the predominance of formal education settings also raises questions about context fit, transferability and the broader scope of CHL promotion. Community-based, healthcare organizational, system-level and policy-oriented interventions were comparatively rare, suggesting that CHL remains largely framed as an educational task directed at individuals rather than a broader societal or institutional responsibility. This may limit the transformative potential of CHL, particularly for populations with limited access to formal education or those facing structural barriers to participation and informed health action.

The conceptual heterogeneity observed across interventions suggests a need for greater transparency regarding the conceptual foundations of CHL interventions. Future studies should report more explicitly which CHL dimensions are addressed and how these are conceptually and operationally defined. Greater conceptual clarity and transparency may facilitate comparability across studies and contribute to the longer-term development of a more coherent understanding of CHL and its intervention approaches.

Evaluation and measurement approaches were heterogeneous, spanning study-specific instruments and established measures. However, none of the approaches captured CHL comprehensively across dimensions, and instruments relying on self-report have been repeatedly criticized because they reflect perceived rather than demonstrated competence and are vulnerable to bias (Steckelberg *et al*. 2017). The field therefore needs CHL-specific outcome concepts and measurement approaches that represent relevant dimensions and allow valid and comparable assessment across contexts.

Future studies should also systematically assess potentially unintended or ambivalent effects of CHL interventions, such as overload, increased uncertainty or interpersonal conflict when CHL competencies are applied in healthcare encounters, particularly in vulnerable groups. Emerging frameworks on the potential adverse effects of interventions to improve critical thinking about health choices may provide a useful starting point for such assessments (Oxman *et al*. 2025).

Most included interventions were evaluated as case studies and pilot studies. This underscores the need to advance interventions along the MRC framework (Skivington *et al*. 2021), including later-phase studies under real-world conditions, process evaluations and implementation studies that explicitly address barriers identified in this review. Longitudinal designs and follow-up assessments are needed to examine retention and transfer into real decision situations over months or even years. Evidence should be expanded for specific target groups, including disadvantaged populations, people with disabilities and people in need of care, in order to better understand feasibility and contextual requirements. Additionally, claims of effectiveness should be interpreted cautiously, as much of the evidence remains exploratory and focused on feasibility or short-term outcomes, with only a few interventions, most notably the IHC programme, having been evaluated in randomized controlled trials including follow-up assessments (I-01–I-05). Moreover, the diversity of evaluation approaches reflects unresolved questions about what CHL interventions are expected to achieve. While cognitive outcomes such as knowledge acquisition, critical appraisal skills or confidence in dealing with health information were commonly assessed, behavioural, social and structural outcomes were addressed less consistently. This imbalance may partly stem from the multi-dimensional nature of CHL itself. Interventions primarily targeting appraisal skills may reasonably be expected to affect proximal cognitive outcomes, whereas interventions addressing informed decision-making, understanding of social determinants or collective action may require evaluation frameworks capable of capturing behavioural change, social participation or broader contextual effects over longer periods of time. Additionally, implementation processes were inconsistently documented across the included interventions. In several studies, facilitators and barriers remained insufficiently described or were not examined systematically. Consequently, the identified barriers and facilitators may reflect selective reporting patterns rather than the full range of implementation experiences across interventions.

Finally, economic evaluation data were rarely reported. For planning, scaling and routine financing, future interventions should prospectively collect and transparently report implementation costs and economic outcomes (e.g. staff time, training effort, materials and infrastructure). Beyond evaluation, future research should sharpen CHL conceptualization in a context-sensitive way to avoid reduction to single subcomponents and to strengthen coherence between theory, intervention design and intervention.

### Strengths and limitations

A strength of this review is the transparent methodology, including an *a priori* protocol registered on OSF (Kalteis *et al*. 2025). Contrary to the protocol’s initial expectation of relatively few inclusions, 81 publications were identified, which is plausible, given a deliberately sensitive search strategy. We used broad terms and synonyms, incorporated adjacent constructs and applied no restrictions regarding population, context, study design or language, aiming for comprehensive coverage. The review process required intensive calibration due to pronounced heterogeneity. To support methodological rigour, uncertainties and disagreements were resolved through structured team discussions, and procedures were aligned with current JBI guidance (Aromataris *et al*. 2024). Search quality was supported through PRESS-based peer review (McGowan *et al*. 2016), and citation searching was conducted and reported in line with TARCiS (Hirt *et al*. 2024). The expertise of the review team in HL and evidence-based health information supported consistent decision-making during screening and extraction.

Limitations should be considered when interpreting the findings. First, conceptual heterogeneity posed a core challenge. Some programmes aligned with CHL dimensions without referencing established CHL definitions, complicating identification and categorical decisions. Although the search strategy was deliberately broad, no specific search component was developed for the CHL dimensions of social determinants of health and collective action. This was because the review primarily focused on CHL in relation to health information, particularly its critical appraisal and use. Accordingly, it remains possible that relevant interventions were missed due to inconsistent terminology or indexing, and conversely that some inclusions would not meet stricter conceptual boundaries. Secondly, grey literature searching was partially constrained by limited access and technical instabilities of some databases, requiring iterative adaptations to search procedures. Thirdly, data extraction evolved iteratively because certain categories were difficult to apply consistently across heterogeneous reports, and studies frequently reported key intervention components incompletely. Finally, given the volume of extracted data, we synthesized findings at a higher level of abstraction, which may have reduced the visibility of more fine-grained details.

## Conclusion

This scoping review provides an overview of interventions aimed at promoting CHL. The evidence base has expanded markedly in recent years and indicates particularly favourable conditions for CHL promotion in educational settings. Schools and higher education offer established infrastructures for early, low threshold and population-wide delivery. The IHC programme provides a useful reference, particularly with regard to iterative development and the provision of ready-to-use teaching materials. However, successful transfer into routine practice will depend on context-sensitive adaptation, deliverer qualification and support, language- and culture-responsive materials and reliable technical and institutional pre-conditions. To strengthen equity and broader societal impact, CHL promotion should increasingly extend beyond formal education settings towards community-based, healthcare organizational and other institutional contexts. The review also highlights important conceptual and practical challenges for future CHL intervention development. Future interventions should operationalize CHL more comprehensively across its dimensions, for example by combining information appraisal and informed decision-making with critical reflection on health inequalities, health system contexts and opportunities for participation or collective action. The review also highlights the risk that conceptual inconsistency may hinder effective implementation of CHL interventions. Realizing CHL’s preventive potential within health promotion will require not only clear conceptual grounding but also strategies that support the translation of competencies into action under real-world social, organizational and informational conditions.

## Supplementary Material

daag103_Supplementary_Data

## Data Availability

The data underlying this article will be shared on reasonable request to the corresponding author.
