# Supplementary material for: Interventions to promote critical health literacy: a scoping review
Source: Health Promot Int. 2026 Aug 5;41(4):daag103. doi: 10.1093/heapro/daag103 (PMC13440308; doi:10.1093/heapro/daag103)
Supplement: Supplementary file 1 [file daag103_supplementary_data.docx]

**Supplementary File 1**

**Search Strings**

MEDLINE via PubMed

| #1 | (health[Title/Abstract] AND "scientific literacy"[Title/Abstract]) OR (health[Title/Abstract] AND "science literacy"[Title/Abstract]) OR "critical health competenc*"[Title/Abstract] OR (critical*[Title/Abstract] AND health[Title/Abstract] AND literacy[Title/Abstract]) OR "health information literacy"[Title/Abstract] |
| --- | --- |
| #2 | intervention*[Title/Abstract] OR strateg*[Title/Abstract] OR program*[Title/Abstract] OR tool*[Title/Abstract] OR project[Title/Abstract] OR trial[Title/Abstract] OR experiment*[Title/Abstract] OR "health education"[Title/Abstract] OR "Health Promotion"[MeSH Terms] OR "health promotion"[Title/Abstract] OR “patient education”[Title/Abstract] OR "Curriculum"[Mesh] OR curricul*[Title/Abstract] OR training[Title/Abstract] |
|  | #1 AND #2 = 2.428 records (26.09.2025) |

CINAHL via EBSCO

| #1 | TI (“health” AND “scientific literacy”) OR AB (“health” AND “scientific literacy”) OR TI (“health” AND “science literacy”) OR AB (“health” AND “science literacy”) OR TI “"critical health competenc*” OR AB "critical health competenc*" OR TI (“critical*” AND health AND literacy) OR AB TI (“critical*” AND health AND literacy) OR TI “health information literacy” OR AB “health information literacy” |
| --- | --- |
| #2 | TI "intervention*" OR AB "intervention*" OR TI "strateg*" OR AB "strateg*" OR TI “program*" OR AB "program*" OR TI "tool*" OR AB "tool*" OR TI "project" OR AB "project" OR TI "trial" OR AB "trial" OR TI "experiment" OR AB "experiment" OR TI "health education OR AB “"health education” OR TI "health promotion" OR AB “"health promotion" OR TI ““patient education” OR AB “patient education” OR TI “curricul*” OR AB “curricul*” OR TI “training” OR AB “training” OR MH "Program Development" OR MH "Teaching Methods" OR MH "Teaching Materials" OR MH "Learning Methods" OR MH "Curriculum" OR MH "Patient Education" OR MH "Health Education" OR MH "Health Promotion" |
|  | #1 AND #2 = 25 records (26.09.2025) |

*Note: Based on a reviewer's comment we rechecked CINAHL search and rerun during revision. No errors in search translation or execution were identified. The comparatively low yield appears to be attributable to limited indexing and use of CHL-specific terminology within the database.*

APA PsycInfo via Ovid

| #1 | (health.ti,ab AND "scientific literacy".ti,ab) OR (health.ti,ab AND "science literacy".ti,ab) OR "critical health competenc*".ti,ab OR (“critical*”.ti,ab AND health.ti,ab AND literacy.ti,ab) OR "health information literacy".ti,ab |
| --- | --- |
| #2 | “intervention*”.ti,ab OR “strateg*”.ti,ab OR program*.ti,ab OR “tool*”.ti,ab OR project.ti,ab OR trial.ti,ab OR “experiment*”.ti,ab OR "health education".ti,ab OR "health promotion".ti,ab OR “patient education”.ti,ab OR “curricul*”.ti,ab OR training.ti,ab OR client education/ OR health education/ OR health knowledge/ OR health promotion/ OR curriculum/ OR curriculum development/ OR learning/ OR learning schedules/ OR learning strategies/ OR learning theory/ |
|  | #1 AND #2 = 807 records (27.09.2025) |

PSYNDEXplus via Ovid

| #1 | (health.ti,ab AND "scientific literacy".ti,ab) OR (health.ti,ab AND "science literacy".ti,ab) OR "critical health competenc*".ti,ab OR (“critical*”.ti,ab AND health.ti,ab AND literacy.ti,ab) OR "health information literacy".ti,ab |
| --- | --- |
| #2 | “intervention*”.ti,ab OR “strateg*”.ti,ab OR program*.ti,ab OR “tool*”.ti,ab OR project.ti,ab OR trial.ti,ab OR “experiment*”.ti,ab OR "health education".ti,ab OR "health promotion".ti,ab OR “patient education”.ti,ab OR “curricul*”.ti,ab OR training.ti,ab OR client education/ OR health education/ OR health knowledge/ OR health promotion/ OR curriculum/ OR curriculum development/ OR learning/ OR learning schedules/ OR learning strategies/ OR learning theory/ |
|  | #1 AND #2 = 29 records (26.09.2025) |

ERIC via IES

| #1 | ((title:health OR abstract:health) AND (title:“scientific literacy” OR abstract:“scientific literacy”)) OR ((title:health OR abstract:health) AND (title:”science literacy" OR abstract:"science literacy")) OR title:"critical health competence" OR abstract:"critical health competence" OR ((title:critical OR abstract:critical) AND (title:health OR abstract:health) AND (title:literacy) OR abstract:literacy)) OR title:"health information literacy" OR abstract:"health information literacy" OR descriptor:"Critical Literacy" |
| --- | --- |
| #2 | title:intervention OR abstract:intervention OR title:strategy OR abstract:strategy OR title:program OR abstract:program OR title:tool OR abstract:tool OR abstract:project OR title:project" OR title:trial OR abstract:trial OR title:experiment OR abstract:experiment OR title:curriculum OR abstract:curriculum OR title:strategy OR abstract:strategy OR title:training OR abstract:training descriptor:"Curriculum" OR title:”health education" OR abstract:"health education" OR descriptor:"Health Education" OR descriptor:"Educational Strategies" OR descriptor:"Training" OR descriptor:"Intervention" |
|  | #1 AND #2 = 295 records (26.09.2025) |

**Seed references**

Table of individual studies (included sources): see *Table 2*.

Table of evidence syntheses with reason for exclusion:

| Author | Year | Title | Reason for exclusion |
| --- | --- | --- | --- |
| Alhussaini et al. | 2025 | Sexual and reproductive health literacy of higher education students: a scoping review of determinants, screening tools, and effective interventions | No relevance to CHL interventions |
| Austvoll-Dahlgren et al. | 2016 | Interventions and assessment tools addressing key concepts people need to know to appraise claims about treatment effects: a systematic mapping review | No relevance to CHL interventions |
| Barbati et al. | 2025 | Effectiveness of ehealth literacy interventions: a systematic review and meta-analysis of experimental studies | No relevance to CHL interventions |
| Barbazi et al. | 2025 | Exploring health educational interventions for children with congenital heart disease: scoping review | No relevance to CHL interventions |
| Barry et al. | 2013 | Interventions for improving population health literacy: insights from a rapid review of the evidence | No relevance to CHL interventions |
| Car et al. | 2011 | Interventions for enhancing consumers’ online health literacy | No relevance to CHL interventions |
| Cusack et al. | 2018 | Educational interventions to improve people’s understanding of key concepts in assessing the effects of health interventions: a systematic review | No relevance to CHL interventions |
| Estrela et al. | 2025 | Educational interventions for the adoption of healthy lifestyles and improvement of health literacy: a systematic review | No relevance to CHL interventions |
| Fernández-González et al. | 2019 | Effective interventions to improve the health literacy of cancer patients | No relevance to CHL interventions |
| Fernández-Gutiérrez et al. | 2018 | Health literacy interventions for immigrant populations: a systematic review | No relevance to CHL interventions |
| Mörelius et al. | 2021 | Digital interventions to improve health literacy among parents of children aged 0 to 12 years with a health condition: systematic review | No relevance to CHL interventions |
| Nutbeam et al. | 2018 | Improving health literacy in community populations: a review of progress | No relevance to CHL interventions |
| Prokop-Dorner, et al. | 2024 | Teaching methods for critical thinking in health education of children up to high school: a scoping review | No relevance to CHL interventions |
| Romanova et al. | 2024 | Improving health and scientific literacy in disadvantaged groups: a scoping review of interventions | No relevance to CHL interventions |
| Rosário et al. | 2024 | Efficacy of health literacy interventions aimed to improve health gains of higher education students-a systematic review | No relevance to CHL interventions |
| Rueskov et al. | 2025 | Health literacy among socioeconomically disadvantaged adolescents: a systematic review of interventions in schools | No relevance to CHL interventions |
| Sardareh et al. | 2024 | Interventions for improving health literacy among older people: a systematic review | No relevance to CHL interventions |
| Sarr, A. et al. | 2025 | Promoting health literacy among migrant populations: implications for adult literacy education | No relevance to CHL interventions |
| Simões et al. | 2024 | Health literacy for elderly patients with high blood pressure: a scoping review | No relevance to CHL interventions |
| Stormacq et al. | 2020 | Effects of health literacy interventions on health-related outcomes in socioeconomically disadvantaged adults living in the community: a systematic review | No relevance to CHL interventions |
| Visscher et al. | 2018 | Evidence on the effectiveness of health literacy interventions in the EU: a systematic review | No relevance to CHL interventions |

**List of excluded articles with reason:**

| Author | Year | Title | Reason for exclusion |
| --- | --- | --- | --- |
| Aghazadeh et al. | 2020 | ’Health literacy for all’: exploring the feasibility of an intervention to reduce health disparities among rural children | No relevance to CHL interventions |
| Alcéna-Stiner et al. | 2020 | The life sciences learning center: an evolving model for a sustainable stem outreach program | No relevance to CHL interventions |
| Andrlová et al. | 2011 | Health information literacy: focusing on patients’ information literacy development | No relevance to CHL interventions |
| Arseneau et al. | 2024 | Points de vue de scientifiques qui agissent sur les plans sociopolitique, scientifique et/ou éducatif dans le contexte de questions scientifiques socialement vives environnementales ou sanitaires | No relevance to CHL interventions |
| Atkinson et al. | 2023 | ’So what are you going to eat?’: insights from a simple foods and intuitive eating workshop series | No relevance to CHL interventions |
| Austvoll-Dahlgren et al. | 2016 | Interventions and assessment tools addressing key concepts people need to know to appraise claims about treatment effects: a systematic mapping review | No relevance to CHL interventions |
| Ayre et al. | 2024 | Online plain language tool and health information quality: a randomized clinical trial | No relevance to CHL interventions |
| Baier et al. | 2015 | Using qualitative methods to create a home health web application user interface for patients with low computer proficiency | No relevance to CHL interventions |
| Barwood et al. | 2020 | Pre-service teachers’ mobilising health literacy in sun safety education | No relevance to CHL interventions |
| Bastos et al.. | 2016 | Comunicação pública da ciência e health literacy no portal da anvisa | No relevance to CHL interventions |
| Bergeron et al. | 2019 | Context and the mechanisms in intersectoral school-based health promotion interventions: a critical interpretative synthesis | No relevance to CHL interventions |
| Betrian et al. | 2023 | Expanding the role of village malaria workers in cambodia: implementation and evaluation of four health education packages | No relevance to CHL interventions |
| Bickham et al. | 2012 | Effects of a media literacy program in the us on children’s critical evaluation of unhealthy media messages about violence, smoking, and food | No relevance to CHL interventions |
| Blanson et al. | 2008 | Usability of an adaptive computer assistant that improves self-care and health literacy of older adults | No relevance to CHL interventions |
| Brown et al. | 2023 | Social media reflection assignment: a simple classroom intervention to help students examine scientific claims in social media | No relevance to CHL interventions |
| Campos et al. | 2020 | Pensamento crítico e jogos digitais: uma análise do jogo plague inc e das narrativas pandêmicas de seus jogadores | No relevance to CHL interventions |
| Chen et al. | 2011 | The role of media literacy and pro-health entertainment programs in changing adolescents’ perceptions of alcohol and alcohol advertising | No relevance to CHL interventions |
| Chen et al. | 2025 | Assessing the effectiveness of interactive robot-assisted virtual health coaching for health literacy and disease knowledge of patients with chronic kidney disease: quasi-experimental study | No relevance to CHL interventions |
| Chesire et al. | 2022 | Contextualizing critical thinking about health using digital technology in secondary schools in Kenya: a qualitative analysis | No relevance to CHL interventions |
| Crafoord et al. | 2023 | Patients’ individualized care perceptions and health literacy using an interactive app during breast and prostate cancer treatment: two parallel randomized controlled trials | No relevance to CHL interventions |
| D’Agostino et al. | 2018 | Public health education: teaching epidemiology in high school classrooms | No relevance to CHL interventions |
| Dai et al. | 2025 | Developing a media literacy-based e-cigarette education program via medical record systems | No relevance to CHL interventions |
| Ernest et al. | 2000 | South Dakota health education standards: a resource guide for achieving health literacy | No relevance to CHL interventions |
| Estacio et al. | 2013 | Health literacy and community empowerment: it is more than just reading, writing and counting | No relevance to CHL interventions |
| Etowa et al. | 2025 | Use of a peer equity navigator intervention to increase access to covid-19 vaccination among African, Caribbean and black communities in Canada | No relevance to CHL interventions |
| Etowa et al. | 2025 | The development and implementation of an innovative community-based intervention to strengthen community capacity for HIV prevention and care among Canadians of African Caribbean and black (acb) women in Ontario: a research protocol | No relevance to CHL interventions |
| Eugenio-Gozalbo et al. | 2022 | Implementing citizen science programmes in the context of university gardens to promote pre-service teachers’ scientific literacy: a study case on soil | No relevance to CHL interventions |
| Evans et al. | 2024 | A global perspective on incorporating health literacy modules into college curriculum | No relevance to CHL interventions |
| Fage-Butler et al. | 2019 | Challenging violence against women: a Scottish critical health literacy initiative | No relevance to CHL interventions |
| Fairbrother et al. | 2020 | Children’s learning from a "smoke-free sports" programme: implications for health education | No relevance to CHL interventions |
| Flores Uribe et al. | 2025 | Overview on the development and application of the key concepts for decision-making in health care | No relevance to CHL interventions |
| George et al. | 2013 | Using animation as an information tool to advance health research literacy among minority participants | No relevance to CHL interventions |
| Göransson et al. | 2020 | An app for supporting older people receiving home care - usage, aspects of health and health literacy: a quasi-experimental study | No relevance to CHL interventions |
| Harvard-Hinchberger et al. | 2006 | Using innovative strategies to enhance health promotion critical literacy | No relevance to CHL interventions |
| Howard et al. | 2017 | Health literacy program proposal for health care workers | No relevance to CHL interventions |
| Israel et al. | 2019 | Does the use of mind mapping as a learning strategy by physician assistant students promote critical thinking as measured by the health science reasoning test? | No relevance to CHL interventions |
| Jakobovich et al. | 2023 | Developing healthy lifestyle behaviors in early age-an intervention study in kindergartens | No relevance to CHL interventions |
| Kahar et al. | 2025 | A community-based health education program: effects and differences in health literacy and self-efficacy across demographic and health variables among Haitians | No relevance to CHL interventions |
| Kinsley et al. | 2009 | A case study for teaching information literacy skills | No relevance to CHL interventions |
| Kite, Lindsay et al. | 2014 | Healthy media literacy: bridging critical media literacy and health literacy to promote positive body image and health | No relevance to CHL interventions |
| Knibbe M et al. | 2017 | Responsible use of media supports ’critical public health literacy’ | No relevance to CHL interventions |
| Kohli et al. | 2023 | Promoting community health and understanding of science topics by reading graphic-style stories aloud together | No relevance to CHL interventions |
| Li et al. | 2022 | Community-based intervention via wechat official account to improve parental health literacy among primary caregivers of children aged 0 to 3 years: protocol for a cluster randomized controlled trial | No relevance to CHL interventions |
| Lindsjö et al. | 2023 | Migrant women’s engagement in health-promotive activities through a women’s health collaboration | No relevance to CHL interventions |
| Long et al. | 2009 | The potential of complementary and alternative medicine in promoting well-being and critical health literacy: a prospective, observational study of shiatsu | No relevance to CHL interventions |
| Lounsbery et al. | 2022 | Beginning early: reflective practice development in a pre-health course on health literacy and health disparities | No relevance to CHL interventions |
| Macauda et al. | 2022 | Elements for successful implementation of a clinic-based health literacy intervention | No relevance to CHL interventions |
| Martin et al. | 2021 | Don’t mention the diet! A health promotion initiative to support healthy diet and lifestyle decision-making by people with intellectual disability | No relevance to CHL interventions |
| McDermott | 2011 | The school health education study + 50 years: scholars’ reflections on its impact and legacy | No relevance to CHL interventions |
| McGeough et al. | 2017 | mHealth and empowerment education in the rainbow nation: a study of the chat program in South Africa | No relevance to CHL interventions |
| McKenna et al. | 2018 | A qualitative study of the development of health literacy capacities of participants attending a community-based cardiovascular health programme | No relevance to CHL interventions |
| Murray et al. | 2022 | The nine cancer frames: a tool to facilitate critical reading of cancer-related information | No relevance to CHL interventions |
| Nittayathammakul et al. | 2022 | Crowd context-based learning process via IoT wearable technology to promote digital health literacy | No relevance to CHL interventions |
| O’Neil et al. | 2018 | Critical appraisal worksheets for integration into an existing small-group problem-based learning curriculum | No relevance to CHL interventions |
| Pardasani et al. | 2018 | Age-tastic! An evaluation of an evidence-based intervention for older adults | No relevance to CHL interventions |
| Phipps et al. | 2018 | Project escuche: a Spanish-language radio-based intervention to increase science literacy | No relevance to CHL interventions |
| Porr et al.. | 2006 | Health literacy as an empowerment tool for low-income mothers | No relevance to CHL interventions |
| Ramírez‐Andreotta et al. | 2023 | Co-created environmental health science: identifying community questions and co-generating knowledge to support science learning | No relevance to CHL interventions |
| Raphael et al. | 2019 | Assuming policy responsibility for health equity: local public health action in Ontario, Canada | No relevance to CHL interventions |
| Ratnayake et al. | 2023 | Investigating anti-vaccination stances on social media: an assignment to promote science literacy | No relevance to CHL interventions |
| Reddy et al. | 2025 | Evaluating gains in student self-efficacy in scientific literacy associated with a brief curricular intervention | No relevance to CHL interventions |
| Renwick et al. | 2014 | Critical health literacy: shifting textual-social practices in the health classroom | No relevance to CHL interventions |
| Renwick et al. | 2017 | Same same but different: curriculum representations of health education | No relevance to CHL interventions |
| Rubinelli et al. | 2021 | Strengthening critical health literacy for health information appraisal: an approach from argumentation theory | No relevance to CHL interventions |
| Sakai H et al. | 2024 | Effectiveness of art-based health education on anaemia and health literacy among pregnant women in western Nepal: a randomized controlled trial | No relevance to CHL interventions |
| Salla et al. | 2010 | Fumo passivo: das concepções dos alunos à utilização de uma ferramenta pedagógica sobre o tema no ensino fundamental: passive smoking: the conceptions of students and the use of a pedagogical tool about the topic in elementary school | No relevance to CHL interventions |
| Salvador et al. | 2018 | Conhecimentos geocientíficos e práticas pedagógicas em ciências naturais na formação inicial de pedagogos | No relevance to CHL interventions |
| Sarvary et al. | 2023 | A multistep science literacy training framework in an introductory biology classroom: teaching how to find, evaluate, comprehend, and cite scientific evidence | No relevance to CHL interventions |
| Shadick et al. | 1993 | Development of a transcultural health education program for the Hmong | No relevance to CHL interventions |
| Shah et al. | 2023 | Remote versus in-person health education: feasibility, satisfaction, and health literacy for diverse older adults | No relevance to CHL interventions |
| Shannon et al. | 2022 | Digital storytelling within the Australian mining industry: worker engagement and health literacy indicator effects | No relevance to CHL interventions |
| Shipman et al. | 2009 | The health information literacy research project | No relevance to CHL interventions |
| Shreffler-Grant et al. | 2021 | Community-based skill building intervention to enhance health literacy among older rural adults | No relevance to CHL interventions |
| Simonds et al. | 2019 | Guardians of the living water: using a health literacy framework to evaluate a child as change agent intervention | No relevance to CHL interventions |
| Smeaton et al. | 2023 | Teaching health literacy and digital literacy together at university level: the flourish module | No relevance to CHL interventions |
| Sommers et et al. | 2025 | Building trust and equity in vaccine communication through community engagement | No relevance to CHL interventions |
| Soriguera et al. | 2023 | La influència de la percepció del so ambiental en l’àmbit educatiu: avaluació, mesures i aplicacions | No relevance to CHL interventions |
| Stone et al. | 2021 | Developing health literacy communication practices for medical students | No relevance to CHL interventions |
| Storrs et al. | 2007 | Critical literacy among the working poor: individualism and pseudostructural interpretive narratives of health inequalities | No relevance to CHL interventions |
| Sundberg et al. | 2021 | Supporting health literacy using an interactive app for symptom management during radiotherapy for prostate cancer | No relevance to CHL interventions |
| Swanberg et al. | 2022 | A health education outreach partnership between an academic medical library and public library: lessons learned before and during a pandemic | No relevance to CHL interventions |
| Świątkiewicz-Mośny et al. | 2022 | When Peppa Pig and Confucius meet, joining forces on the battlefield of health literacy - a qualitative analysis of Covid-19 educational materials for children and adolescents from China, the USA, and Europe | No relevance to CHL interventions |
| Takahashi et al. | 2022 | Effect of different educational interventions on knowledge of HPV vaccination and cervical cancer among young women: a preliminary report | No relevance to CHL interventions |
| Takahashi et al. | 2025 | Effects of different educational interventions on cervical cancer knowledge and human papillomavirus vaccination uptake among young women in Japan: preliminary results of a cluster randomized controlled trial | No relevance to CHL interventions |
| Turner et al. | 2016 | Identification of health literacy competencies by nurse educators in an associate degree nursing program | No relevance to CHL interventions |
| Ussher et al. | 2021 | A randomized controlled evaluation of an educational resource to address fertility concerns after cancer | No relevance to CHL interventions |
| Vandendriessche et al. | 2018 | 2bic: taking your adventuring gear to organise pop-up health information literacy sessions | No relevance to CHL interventions |
| Wang et al. | 2016 | Patient empowerment interacts with health literacy to associate with subsequent self-management behaviors in patients with type 2 diabetes: a prospective study in Taiwan | No relevance to CHL interventions |
| Wang et al. | 2025 | The impact of evidence-based medicine curricula on information literacy among clinical medical undergraduates and postgraduates in China | No relevance to CHL interventions |
| Warren et al. | 2019 | Action groups as a participative strategy for leading whole-school health promotion: results on implementation from the inclusive trial in English secondary schools | No relevance to CHL interventions |
| Witherly | 2010 | Instituting a standards-based K‐12 science curriculum supplement program at the national institutes of health: a case study | No relevance to CHL interventions |
| Woods-Townsend et al. | 2021 | A cluster-randomised controlled trial of the lifelab education intervention to improve health literacy in adolescents | No relevance to CHL interventions |
| Woods-Townsend et al. | 2018 | Lifelab Southampton: a programme to engage adolescents with DOHaD concepts as a tool for increasing health literacy in teenagers - a pilot cluster-randomized control trial | No relevance to CHL interventions |
| Wu et al. | 2021 | Health promotion in the community via an intergenerational platform: intergenerational e-health literacy program (I-Help) | No relevance to CHL interventions |
| Zanchetta et al. | 2023 | Critical education in community health literacy for Brazilian nurses: a course evaluation | No relevance to CHL interventions |
| Zanchetta et al. | 2014 | Awakening professionals’ critical awareness of health literacy issues within a francophone linguistic-minority population in Ontario | No relevance to CHL interventions |
| Zhou et al. | 2025 | The effectiveness of empowerment versus fear messaging on sugary beverage consumption intentions among young adult Latinas | No relevance to CHL interventions |
| Zwickey et al. | 2014 | Teaching evidence-based medicine at complementary and alternative medicine institutions: strategies, competencies, and evaluation | No relevance to CHL interventions |
